# Characterization of long non-coding RNA-associated ceRNA network to reveal potential prognostic lncRNA biomarkers in human ovarian cancer

**DOI:** 10.18632/oncotarget.7181

**Published:** 2016-02-03

**Authors:** Meng Zhou, Xiaojun Wang, Hongbo Shi, Liang Cheng, Zhenzhen Wang, Hengqiang Zhao, Lei Yang, Jie Sun

**Affiliations:** ^1^ College of Bioinformatics Science and Technology, Harbin Medical University, Harbin, PR China

**Keywords:** biomarker, competing endogenous RNA, long non-coding RNA, ovarian cancer

## Abstract

Accumulating evidence has underscored the important roles of long non-coding RNAs (lncRNAs) acting as competing endogenous RNAs (ceRNAs) in cancer initiation and progression. In this study, we used an integrative computational method to identify miRNA-mediated ceRNA crosstalk between lncRNAs and mRNAs, and constructed global and progression-related lncRNA-associated ceRNA networks (LCeNETs) in ovarian cancer (OvCa) based on “ceRNA hypothesis”. The constructed LCeNETs exhibited small world, modular architecture and high functional specificity for OvCa. Known OvCa-related genes tended to be hubs and occurred preferentially in the functional modules. Ten lncRNA ceRNAs were identified as potential candidates associated with stage progression in OvCa using ceRNA-network driven method. Finally, we developed a ten-lncRNA signature which classified patients into high- and low-risk subgroups with significantly different survival outcomes. Our study will provide novel insight for better understanding of ceRNA-mediated gene regulation in progression of OvCa and facilitate the identification of novel diagnostic and therapeutic lncRNA ceRNAs for OvCa.

## INTRODUCTION

MicroRNAs (miRNAs) are a major class of short non-coding RNAs (ncRNAs) with ∼20 nucleotides in length, and participate in a wide range of biological processes [1]. miRNAs can regulate gene expression at the post-transcriptional level through binding to miRNA response elements (MREs) on the 3′ untranslated region (3′UTR), coding sequence (CDS) and 5′UTR of target gene [2]. It has been shown that diverse RNA molecules harboring MREs can act as competing endogenous RNAs (ceRNAs) to communicate by competing for a common pool of miRNAs, leading to the ‘ceRNA hypothesis' [3, 4]. CeRNA crosstalk represents an exciting novel layer of miRNA regulatory network and forms complex miRNA-mediated ceRNA networks (ceRNETs). There is increasing evidence shown that ceRNA crosstalk occurs widely in essential cellular processes and functions, and its perturbation will disrupt the balance of the ceRNETs leading to disease initiation and progression [4, 5].

Long non-coding RNAs (lncRNAs), a newly described subclass of ncRNAs, was arbitrarily defined as ncRNAs of larger than 200 nucleotides in length distinguished from short ncRNAs [6]. A growing body of evidence has shown that lncRNAs function as a crucial component of complex gene regulatory network by regulating gene expression at the transcriptional, post-transcriptional and epigenetic levels [7, 8]. Recent theoretical and experimental studies have demonstrated the ceRNA activity of lncRNAs as natural miRNA decoys in human development and pathophysiological conditions [9]. Systematic analysis of lncRNA-associated ceRNA network have been performed in breast cancer [10, 11], gastric cancer [12] and glioblastoma multiforme [13]. A more recent study reported lncRNA *HOST2* as miRNA *let-7b* sponge to inhibit *let-7b* functions, thereby contributing to ovarian cancer (OvCa) [14], revealing the functional significance of lncRNA-associated ceRNA network in OvCa for the first time. However, the complexity and behavior of lncRNA-associated ceRNA network remains poorly characterized in the progression of OvCa.

Here, we used an integrative computational method to identify miRNA-mediated ceRNA crosstalk between lncRNAs and mRNAs, and reconstructed global and progression-related lncRNA-associated ceRNA networks (LCeNETs) with sample-matched miRNA, mRNA and lncRNA expression profiles of 401 OvCa patients with stage I, III and IV derived from TCGA based on “ceRNA hypothesis”. We identified key lncRNAs associated with distinct stages of OvCa progression using a ceRNA-network driven method, and developed a ten-lncRNA signature to predict the clinical outcome of OvCa. The methodology presented seems to be the first implementation of progression-related ceRNA network to identify candidate prognostic lncRNA biomarkers.

## RESULTS

### Global properties and functional characterization of OvCa-specific LCeNET

**Figure 1:**
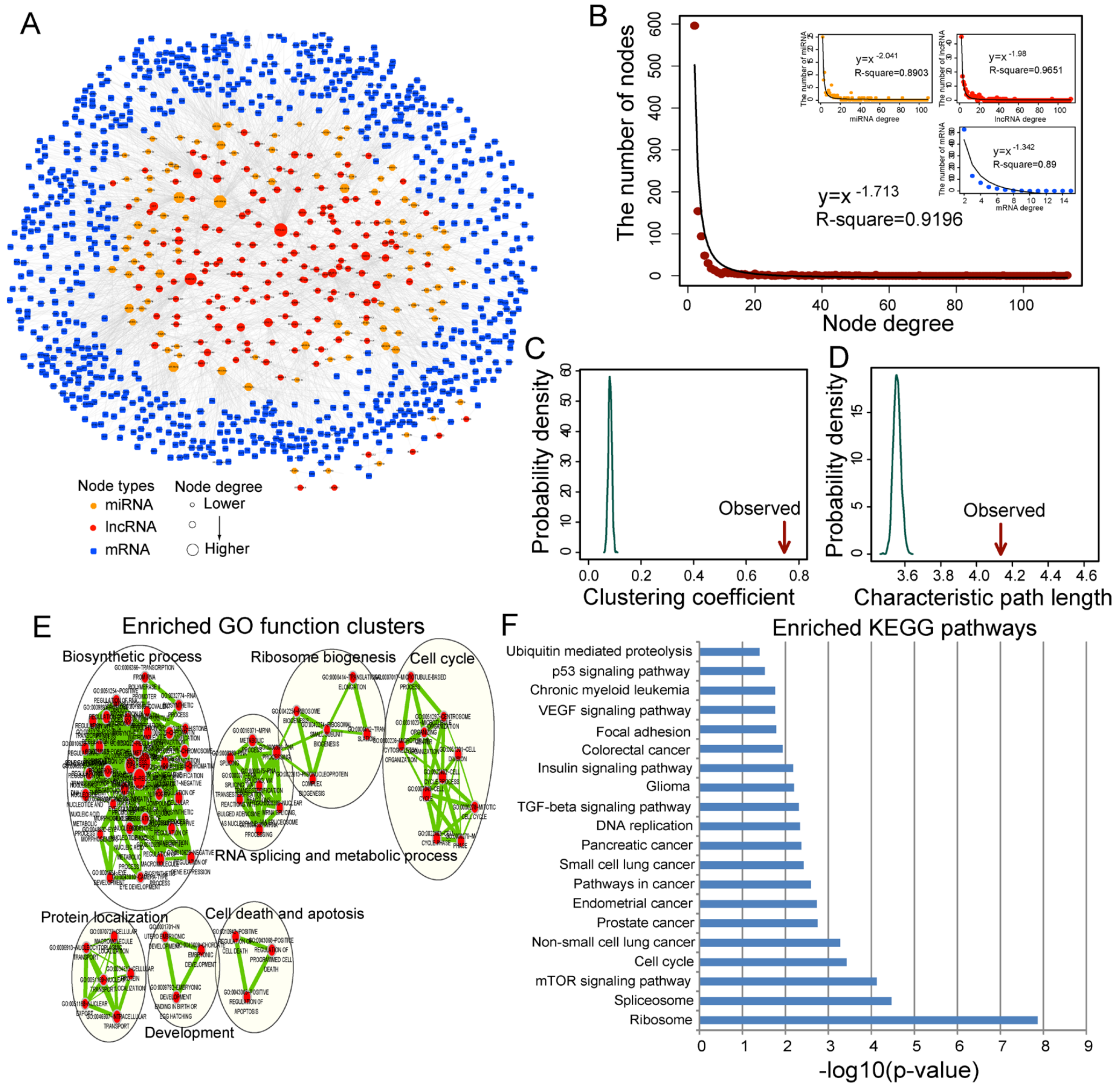
Ovarian cancer-specific lncRNA-associated ceRNA network and their characteristics **A.** Global view of the LCeNET in ovarian cancer. This network consists of 1045 nodes and 2516 links. **B.** Degree distribution of the LCeNET. **C.** The clustering coefficient of the LCeNET is higher than randomization test. The arrow represents the clustering coefficient in the real network. **D.** The characteristic path length of the LCeNET is higher than randomization test. The arrow represents the characteristic path length in the real network. **E.** The functional enrichment map of GO terms. Each node represents a GO term, which are grouped and annotated by GO similarity. A link represents the overlap of shared genes between connecting GO terms. Node size represents the number of gene in the GO terms. Color intensity is proportional to enrichment significance. **F.** Significantly enriched KEGG pathway of mRNAs in the LCeNET.

We integrated matched expression profiles of 401 OvCa patients from TCGA and experimentally validated interaction network among miRNAs, mRNAs and lncRNAs to identify functional miRNA-mediated LMceCTs. As described in the Methods section, a total of 1270 miRNA-mediated ceRNA crosstalk between lncRNAs and mRNAs (LMceCTs) were identified (Supplementary File 1). Then these functional LMceCTs were integrated to build a global OvCa-specific LCeNET. The constructed LCeNET contained 1045 nodes (including 97 miRNAs, 150 lncRNAs and 798 mRNAs) and 2516 edges (Figure 1A). To explore the architecture and features of OvCa-specific LCeNET, network analysis was performed and the results were summarized in Table 1. As observed, the degree distribution of nodes in the LCeNET closely followed a power law distribution with *R*^2^=0.9196 (Figure 1B). Most nodes had relatively few interactions with others and only a small portion of nodes had a large number of interactions. The topology analysis suggested that the LCeNET had a small-world organization with high small-world index of 7.779 and high clustering coefficient of 0.745 (empirical *p* < 0.001) (Figure 1C) compared with random networks. However, it is interesting to observe that the characteristic path length is slightly larger than random networks which may be due to the lack of extremely long-range connections (Figure 1D).

**Table 1:** Network characteristics of OvCa-specific and progression-related LCeNETs

	OvCa-specific LCeNE	Stage II-related LCeNET	Stage III-related LCeNET	Stage IV-related LCeNET
Number of nodes	1045	1114	1180	839
Number of edges	2516	2391	2837	2046
Clustering coefficient	0.745	0.768	0.748	0.735
Characteristic path length	4.144	4.418	4.038	4.074
Small world property	7.779	11.829	7.332	8.076
Average number of neighbors	4.815	4.293	4.842	4.877
Connected components	7	7	3	6
Network diameter	10	10	8	9
Network radius	1	1	1	1
Network density	0.005	0.004	0.004	0.006
Network Heterogeneity	1.877	1.853	2.018	1.730

To further validate potential functional implication of LCeNET in OvCa, we performed functional enrichment analysis of mRNAs in the LCeNET based on Gene Ontology (GO) and Kyoto Encyclopedia of Genes and Genomes (KEGG) pathways, and found that these mRNAs were significantly enriched in 211 GO terms (*p* < 0.05 and Fold Enrichment > 1.5) mainly involved in six functional clusters including RNA splicing, biosynthetic process, cell death and apoptosis, cell cycle, morphogenesis and development and mRNA catabolic process (Figure 1E), and 20 KEGG pathways including pathways in cancer, ribosome pathway and several signaling pathways (Figure 1F) (Supplementary File 2). All the enriched signaling pathways, including mTOR signaling pathway, TGF-beta signaling pathway, Insulin signaling pathway, VEGF signaling pathway and p53 signaling pathway, are well known to contribute to the pathogenesis of OvCa [15-18]. These results suggested that lncRNA-associated ceRNA regulation in the LCeNET participated in broad biological functions associated with OvCa.

### Hub nodes in the LCeNET play critical roles in OvCa

**Figure 2:**
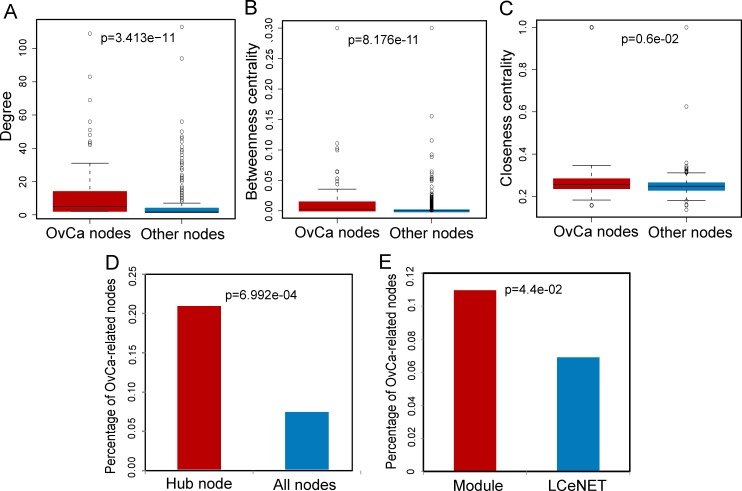
The ovarian cancer-associated nodes tend to be hubs and are enriched in modules **A.** The difference of degree between ovarian cancer-associated nodes and other nodes. Ovarian cancer-associated nodes had a higher degree than other nodes. **B.** The difference of betweenness centrality between ovarian cancer-associated nodes and other nodes. Ovarian cancer-associated nodes had a higher betweenness centrality than other nodes. **C.** The difference of clustering coefficient between ovarian cancer-associated nodes and other nodes. Ovarian cancer-associated nodes had a higher clustering coefficient than other nodes. *P*-values were calculated based on Wilcoxon rank sum test. **D.** The proportion of ovarian cancer-associated nodes among hubs and all nodes in the LCeNET. **E.** The proportion of ovarian cancer-associated nodes among modules and LCeNET. *P*-values were calculated based on Hypergeometric test.

We mapped known OvCa-related genes to the LCeNET, and found that known OvCa-related genes were significantly enriched in the LCeNET (*p* < 0.001, Hypergeometric test). Further network analysis revealed significantly different topological characteristics between known OvCa-related nodes and other nodes in the LCeNET. The OvCa-related nodes have significantly higher degrees, betweenness centrality and closeness centrality than other nodes in the LCeNET (avg. 13.831 *vs*. 4.158 for degrees, *p* = 3.413e-11, Figure 2A; avg. 0.018 *vs*. 0.003 for betweenness centrality, *p* = 8.176e-11, Figure 2B; avg. 0.296 *vs*. 0.256 for closeness centrality, *p* = 0.006, Figure 2C; Wilcoxon rank sum test), implying that hub nodes in the LCeNET were far more important than non-hub nodes, and were more likely associated with OvCa.

To determine the hub nodes in the LCeNET, all nodes in the LCeNET were sorted in a descending order according to their degree. We chose the top 5 percent of miRNAs, lncRNAs and mRNAs with the highest degree as the hub components according recent studies [19, 20]. We identified 5 hub miRNAs (HubmiRs), 8 hub lncRNAs (HublncRs) and 40 hub mRNAs (HubmRs), and found that known OvCa-related genes were significantly enriched in the hubs (*p* = 6.992e-04, Hypergeometric test) (Figure 2D). 11 of 53 hub nodes were known OvCa-related genes, including 5 HubmiRs, 1 HublncR and 5 HubmRs. All of these observations demonstrated that hub nodes in the LCeNET were significantly more likely to be essential for OvCa development and progression compared with non-hub nodes.

We further investigated the modularity feature of the LCeNET. Based on the OvCa-specific LCeNET, 16 OvCa-related functional modules, comprising 129 genes, were identified using molecular complex detection (MCODE) method [21]. These functional modules were numbered from 1 to 16 in order of decreasing module size (Supplementary File 3). We found that these functional modules varied greatly in size, ranging from 3 to 58 genes, with a mean size of 16 genes. Known OvCa-related genes in the LCeNET were observed to occur preferentially in these functional modules (*p* = 0.044, Hypergeometric test), suggesting that these functional modules were significantly associated with OvCa. We also found a hub miRNA *miR-186-5p* function as a date hub to connect four functional modules (module 2, 3, 4 and 5), implying its important roles in organizing the functional modules. A recent study suggested that *miR-186* can act as a key player in overcoming chemoresistance in ovarian cancer therapy [22], which strongly supported our findings.

### Progression-related network analysis reveals prognostic lncRNA biomarkers associated with progression of OvCa

To identify potential prognostic lncRNA biomarkers associated with progression of OvCa stages, we further constructed progression-related LCeNETs of OvCa patients in stage II, III and IV based on correlated relationships among miRNAs, lncRNAs and mRNAs under a specified condition. The stage II and III-related LCeNETs have significantly more nodes and edges than the stage IV-related LCeNET. The stage II-related LCeNET contains 1114 nodes (101 miRNAs, 204 lncRNAs and 809 mRNAs) and 2391 edges, and stage III-related LCeNET contains 1180 nodes (99 miRNAs, 162 lncRNAs and 919 mRNAs) and 2837 edges, whereas stage IV-related LCeNET contains only 839 nodes (88 miRNAs, 139 lncRNAs and 612 mRNAs) and 2046 edges (Supplementary File 4). Network analyses revealed that all three progression-related LCeNETs had similar topological properties, such as the clustering coefficient (0.768, 0.748 and 0.735, respectively), characteristic path length (4.418, 4.038 and 4.074, respectively) and small world index (11.829, 7.332 and 8.076, respectively) (Table 1 and Supplementary File 5). In order to identify potential critical lncRNAs associated with OvCa progression, we focused our attention on hub lncRNAs (are hereafter referred to as HublncR) in progression-related LCeNETs.

We identified HublncR as the top 5% with the highest degree for lncRNAs, and 10, 8 and 7 lncRNAs were identified as HublncRs in three progression-related LCeNETs respectively (Supplementary File 6). One of HublncRs, *NEAT1* was commonly shared among three progression-related LCeNETs, implying that *NEAT1* was most likely to play an important role in OvCa. Although several previous studies have reported the important roles of *NEAT1* as biomarker in acute promyelocytic leukemia [23] and prostate cancer [24], little is known about the role of lncRNA *NEAT1* in OvCa. More recently, a latest study through cell proliferation assays and migration assays performed by Patel *et al*. found OvCa cell migration decreased when lncRNA *NEAT1* was silenced [25], which provided experimental evidences for functional implication of *NEAT1* in OvCa. Three lncRNAs (*TP73-AS1*, *AC000120.7* and *CTB-89H12.4*) were identified as HublncRs both in stage III and IV-related LCeNETs, implying their critical functional roles in the advanced stage of OvCa. lncRNA *TP73-AS1*, the antisense of the protein-coding gene *TP73,* has been reported to be associated with tumorigenesis and histological differentiation and can function as a biomarker in non-small-cell lung carcinomas (NSCLC) [26]. lncRNA *AC000120.7* overlaps with the sense strand of protein-coding gene *KRIT1*. *KRIT1* is a binding partner of the GTPase Rap1a and can function as a tumor suppressor [27]. lncRNA *CTB-89H12.4* is the retained intron of protein-coding gene *CSNK1A1*. Previous study has suggested that the expression of *CSNK1A1* is implicated in advanced stage (III/IV) of OvCa [28]. A recent study about relationships between lncRNAs and protein-coding genes has suggested that the function of lncRNA overlapping with protein-coding gene tended to be similar to this protein-coding gene [29]. These results implied that *AC000120.7* and *CTB-89H12.4* may function by posttranscriptional regulation of the *KRIT1* and *CSNK1A1* genes, and had significant roles in advanced stage (III/IV) of OvCa. Ten HublncRs were found to be stage-specific, including six HublncRs for stage II-specific, two HublncRs for stage III-specific and two HublncRs for stage IV-specific. These stage-specific HublncRs may have important functions in individual stages in the course of OvCa progression.

**Figure 3:**
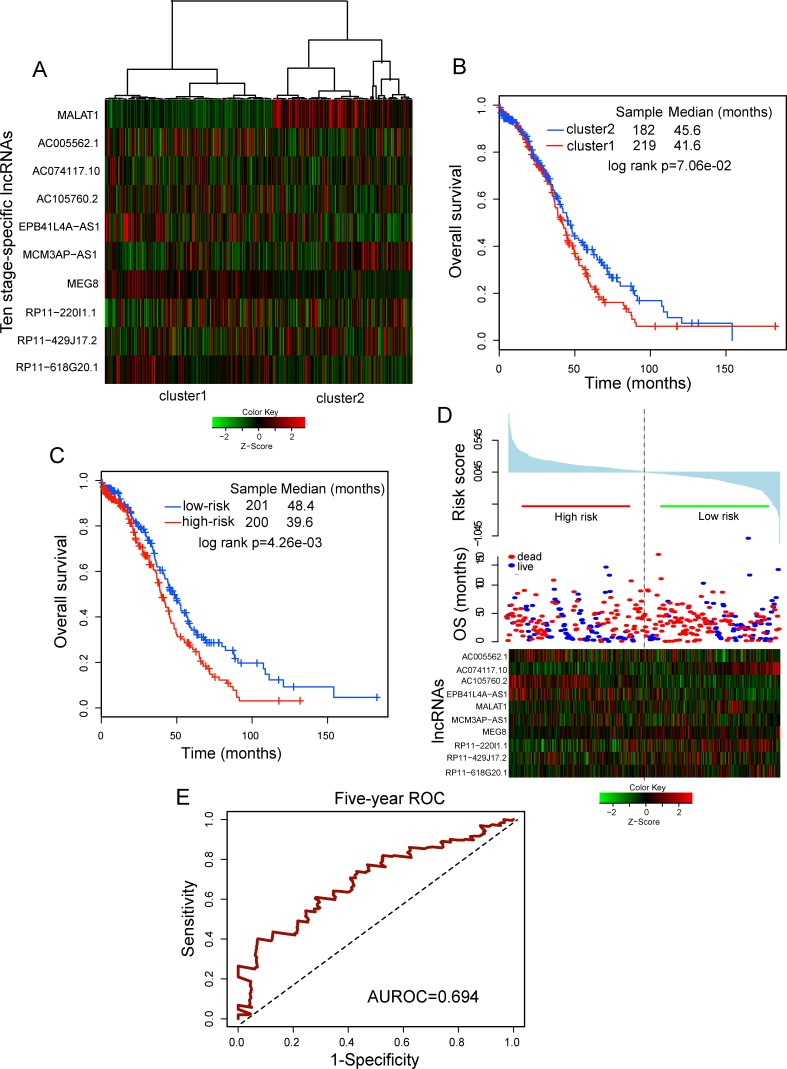
Prognostic value of ten-lncRNA signature for assessing clinical outcome of ovarian cancer **A.** Hierarchical clustering heatmap and dendrogram of ovarian cancer samples based the expression patterns of ten stage-specific HublncRs. **B.** Kaplan-Meier survival curves for ovarian cancer samples classified into two subgroups using the unsupervised hierarchical clustering strategy. P-Values were calculated using the log-rank test. **C.** Kaplan-Meier survival curves for ovarian cancer samples classified into high-risk and low-risk groups using the ten-lncRNA signature. P-values were calculated using the log-rank test. **D.** The ten lncRNA-based risk score distribution, patients' survival status and heatmap of the ten stage-specific HublncRs expression profiles. The black dotted line represents the cutoff value of the risk score derived from the TCGA patients which separated patients into high- and low-risk groups. **E.** Receiver operating characteristic (ROC) analysis of the risk scores for overall survival prediction in the TCGA dataset.

Based on above observations, we further explored whether these ten stage-specific HublncRs had prognostic significance for predicting clinical outcome in OvCa. We used an unsupervised hierarchical clustering strategy to group the expression patterns of ten HublncR and 401 patients with OvCa. All patients were divided into two subgroups (219 patients *vs*. 182 patients) based on the first bifurcation of the clustering dendrogram (Figure 3A). As seen in Figure 3B, survival analysis revealed obvious difference in overall survival (OS) between these two patients subgroups (median OS 41.6 months *vs*.45.6 months) (log-rank test *p* = 7.6E-02; Figure 3B), indicating the prognostic potential of ten stage-specific HublncRs as candidate biomarkers in the prediction of clinical outcomes. Although most of these stage-specific lncRNAs have not been functionally characterized, hub lncRNA *MALAT1* is well known to promote cancer metastasis in lung, colorectal, bladder and multiple myeloma when its expression was up-regulated [30-32]. Moreover, recent study has demonstrated aberrant expression of lncRNA *MALAT1* in OvCa-associated fibroblasts [33], which was consistent with results produced by clustering analysis.

### Prognostic value of ten-lncRNA signature for assessing clinical outcome of OvCa

To build a lncRNA signature to predict survival outcome in OvCa, these ten HublncRs were fitted in a multivariate Cox regression model with OS as the dependent variable and other clinical information as covariables. A ten-HublncR-based risk score model was constructed according to a linear combination of expression values of these ten HublncRs weighted by the regression coefficients derived from multivariate Cox regression analysis as follows: Risk score = (5.303e-02* expression value of *AC005562.1*)+(−8.968e-02* expression value of *AC074117.10*)+(6.192e-01*expression value of *AC105760.2*)+(1.088e-02* expression value of *EPB41L4A-AS1*)+(1.182e-06*expression value of *MALAT1*)+(4.516e-02* expression value of *MCM3AP-AS1*)+(−1.121e-01* expression value of *MEG8*)+(−1.045e-02* expression value of *RP11-220I1.1*)+(5.753e-02* expression value of *RP11-429J17.2*)+(−3.291 * expression value of *RP11-618G20.1*). We then calculated the ten-HublncR signature based risk score for each patient in the TCGA dataset (*n* = 401). The patients were divided into a high-risk group (*n* = 200) and a low-risk group (*n* = 201) using the median risk score as the cut-off. Patients in the high-risk group had significantly shorter survival than those in the low-risk group (median 39.6 months *vs*. 48.4 months, log-rank p = 4.26e-03) (Figure 3C). The 5-year survival rate of the high-risk group was 26.6%, whereas the corresponding rate in the low-risk group was 34.2%. In the univariate analysis, the hazard ratios of low-risk *versus* high-risk group was 2.718 (*p* = 0.002; 95% confidence interval (CI) = 1.458-5.068) (Table 2). The distribution of risk score, patient status and ten HublncR expression in 401 patients of TCGA dataset are shown in Figure 3D. Of these ten HublncRs, four were found to be risky lncRNAs (*AC005562.1*, *AC105760.2*, *EPB41L4A-AS1* and *MCM3AP-AS1*) and six were found to be protective lncRNAs (*AC074117.10*, *MALAT1*, *MEG8*, *RP11-220I1.1*, *RP11-429J17.2* and *RP11-618G20.1*). Patients with high-risk scores tended to express risky HublncRs, whereas patients with low-risk scores tended to express protective HublncRs. Furthermore, we performed the time-dependent ROC curve analysis to evaluate sensitivity and specificity for survival prediction of ten-HublncR signature in the entire TCGA set. As shown in Figure 3E, the ten-HublncR signature achieved AUC values of 0.694, demonstrating its better prediction performance.

**Figure 4:**
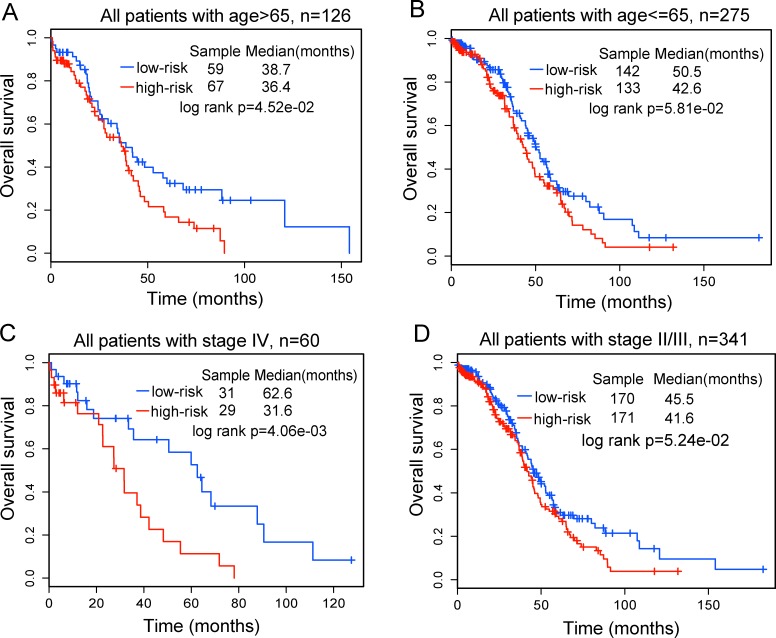
Stratification analyses of all patients with available age or tumor stage information using the ten-lncRNA signature **A.** Kaplan-Meier survival curves for elder patients with OvCa (age > 65, *n* = 126). **B.** Kaplan-Meier survival curves for younger patients with OvCa (age < = 65, *n* = 275). **C.** Kaplan-Meier survival curves for all patients with stage IV (*n* = 60). **D.** Kaplan-Meier survival curves for all patients with II and III (*n* = 341). *P*-values were calculated using the log-rank test.

We further investigated whether the prognostic value of the ten-HublncR signature was independent of other clinical variables. For this, we first performed multivariate Cox regression analysis including ten-HublncR risk score, age, grade, stage and surgical debulking status as covariates. The results showed that ten-HublncR risk score (HR = 2.485, *p* = 0.004), age (HR = 1.018, *p* = 0.007) and stage IV (HR = 2.666, *p* = 0.044) were independent prognostic factors (Table 2). Next, data stratification analysis was then performed for age and stage. All patients of TCGA dataset were stratified by age into either an elder stratum (age > 65) or a younger stratum (age≤65). This stratified analysis showed effective prognostic power of the ten-HublncR signature in both the younger and elder patient groups. The ten-HublncR signature could classify patients within each age stratum into either high- or low-risk groups with significantly different OS (median OS 36.4 months *vs*. 38.7 months, log-rank test *p* = 0.045 for the elder patient group and median OS 42.6 months *vs*. 50.5 months, log-rank test *p* = 0.058 for the younger patient group) (Figure 4A and 4B), indicating that the prognostic power of ten-HublncR signature was also age-independent. Then the patients of stage II-III and stage IV for TCGA dataset were classified into two separate groups. The stratified analysis was further performed in patients group with stage II-III and patients with stage IV to evaluate whether the ten-HublncR signature could predict OS of patients for different clinical stage. The results of stratification analysis showed that the ten-HublncR signature could further subdivide patients with stage IV into either a high-risk group with shorter survival or a low-risk group with longer survival (median OS 31.6 months *vs*. 62.6 months, log-rank test *p* = 0.004) (Figure 4C). Difference for OS between high-risk group (*n* = 171) and low-risk group (*n* = 170) was also observed in patients with stage II-III (median OS 41.6 months *vs*.45.5 months) (Figure 4D), although the log-rank p value is 0.052 which was slightly above the 0.05 significance level.

**Table 2:** Univariate and multivariate Cox regression analysis of the ten-lncRNA signature and overall survival of OvCa patients in the TCGA cohort

Variables		Univariate analysis			Multivariate analysis		
		HR	95% CI of HR	*P*-value	HR	95% CI of HR	*P*-value
Age		1.017	1.005-1.03	0.006	1.018	1.005-1.031	0.007
Stage	II	1 (reference)			Reference		
	III	1.958	0.919-4.269	0.082	2.086	0.842-5.168	0.112
	IV	2.233	0.994-5.017	0.052	2.666	1.027-6.921	0.044
Grade	G1/G2	1 (reference)			Reference		
	G3/G4	1.343	0.912-1.978	0.136	1.388	0.921-2.091	0.117
Residual	0-10mm	1 (reference)			Reference		
	>10mm	1.224	0.914-1.638	0.175	1.129	0.835-1.526	0.430
lncRNA risk score		2.718	1.458-5.068	0.002	2.485	1.328-4.647	0.004

## DISCUSSION

Recently, ceRNA hypothesis has been proposed to represent a novel post-transcriptional layer of gene regulation working through miRNA competition [3, 4]. With the discovery of ceRNA crosstalk, it has been shown that miRNA and their ceRNA targets can connect directly or indirectly to form a complex ceRNA network [5]. In the present study, based on the ceRNA hypothesis, we utilized paired miRNA, lncRNA and mRNA expression profiles of OvCa patients in combination with experimentally validated miRNA-target interactions to reconstruct lncRNA-associated ceRNA network in the progression of OvCa. The constructed OvCa-related LCeNETs provide important clues for understanding the key roles of ceRNA-mediated gene regulatory network in the development and progression of OvCa.

Complex alterations of disease-specific or stage-specific global expression profiles for miRNAs, lncRNAs and mRNAs, and the resultant changes in lncRNA-associated ceRNA crosstalk interactions, may become the determinant of progression of cancer stages [4]. We found that three progression-related LCeNETs exhibited substantial differences in ceRNA crosstalk interactions, even though their network structures were similar. These differences may be attributed to miRNA and ceRNA abundance variations and rewiring interaction in the progression of OvCa. We further investigated the observed variations of stage-specific LCeNETs and found ten stage-specific HublncRs associated with OvCa stage. Based on the expression patterns of these ten stage-specific lncRNA hubs, 401 patients with OvCa were classified into two groups with different clinical outcomes, indicating the potential roles of ten hub lncRNAs as potential prognostic biomarkers for predicting the clinical outcome in OvCa.

The differential expressions of lncRNAs have been widely observed in various cancers [34-37], and their expressional perturbation has been implicated in the development and progression of cancers [38, 39]. Several lncRNA signatures have been developed to improve prognosis prediction of cancers, including colorectal cancer [40], glioblastoma multiforme [41], breast cancer [42], lung cancer [43] and multiple myeloma [44]. Recently, Du and colleagues identified approximately 100 lncRNAs correlated with OS using Cox regression analysis [45]. However, the prognostic role of lncRNA signature in OvCa has not been investigated. So we created a risk score model according to the patients' expression values of ten stage-specific HublncRs, and applied this ten-HublncR signature to the TCGA patients. We found that the ten-HublncR signature was able to differentiate OvCa patients between poor prognosis and good prognosis on the basis of differences in their expression profiles.

From our literature review, we found that one of ten HublncRs, *MALAT1*, was a well-known prognostic marker linked to several cancers [46]. Another lncRNA, *MEG8*, was an imprinted gene which showed preferentially expressed in skeletal muscle [47]. As functional research of lncRNAs is still in its infancy, the functions of remaining eight HublncRs have not been reported yet. Currently, computational prediction for lncRNA function has demonstrated many advantages of the functional interpretation by their co-expressed mRNAs [48, 49]. So, we predicted lncRNA function through GO and KEGG enrichment analysis for its all mRNA neighbors in the ceRNA network, and top one enriched functional annotation of GO term and KEGG pathway was considered as potential function of *lncRNA*. We found that inferred functions of these HublncRs were involved in Hedgehog signaling pathway, VEGF signaling pathway, Wnt receptor signaling pathway, tube development, cell adhesion and ECM-receptor interaction, which are fundamental processes for cancer growth and are relevant to OvCa progression. For example, hedgehog signaling pathway involves in a variety of developmental process and its aberrant activation has profound effect on OvCa progression [50]. Vascular endothelial growth factor (VEGF), a key regulator of angiogenesis, has been implicated in OvCa progression, and VEGF signaling pathway has revealed its value as a therapeutic target in patients with OvCa [51]. A previous study suggested that abnormal activation of Wnt signaling pathway can promotes OvCa progression [52]. The negative effect on ECM-receptor interaction is able to inhibit OvCa progression by reducing invasive activity of cancer cells [53]. Functional analysis has suggested that these ten stage-specific HublncRs played important roles in OvCa and their expression patterns were correlated with distinct stages of OvCa progression. However, further experimental studies should be conducted to uncover the functional roles of these lncRNAs in OvCa progression. To our knowledge, the sample-matched expression profiles of mRNA, miRNA and lncRNAs in OvCa patients derived from TCGA are unprecedented in comprehensiveness. There is no other independent datasets to validate our findings owing to the limitation of available lncRNA expression. This ten-HublncR signature, if validated prospectively, may have important implications for the identification of novel diagnostic and therapeutic lncRNA ceRNAs in OvCa.

**Table 3:** Overall information and predicted functions of ten stage-specific HublncRs

Ensembl id	Ensembl name	Chromosomal position	Known disease	Known function	Top 1 enriched GO function	Top1 enriched KEGG pathway
ENSG00000214719	AC005562.1	Chr17: 30,576,464-30,672,789 (+)	Unknown	Unknown	cellular hormone metabolic process	NA
ENSG00000234072	AC074117.10	Chr2: 27,356,246-27,367,622 (+)	Unknown	Unknown	transcription	NA
ENSG00000227252	AC105760.2	Chr2: 237,059,434-237,085,817 (−)	Unknown	Unknown	limb morphogenesis	Hedgehog signaling pathway
ENSG00000224032	EPB41L4A-AS1	Chr5: 112,160,526-112,164,276 (+)	Unknown	Unknown	translational elongation	Ribosome
ENSG00000251562	MALAT1	Chr11: 65,497,762-65,506,516 (+)	lung, colorectal, bladder, ovarian cancers and multiple myeloma	alternative splicing and cell cycle	regulation of transcription	VEGF signaling pathway
ENSG00000215424	MCM3AP-AS1	Chr21: 46,229,217-46,259,390 (+)	Unknown	Unknown	positive regulation of Wnt receptor signaling pathway	Cysteine and methionine metabolism
ENSG00000258399	MEG8	Chr14: 100,894,770-100,935,999 (+)	Unknown	imprinted gene	tube development	NA
ENSG00000281649	EBLN3	Chr9: 37,079,857-37,090,507 (+)	Unknown	Unknown	transcription	NA
ENSG00000181097	RP11-429J17.2	Chr8: 143,696,154-143,698,413 (+)	Unknown	Unknown	cell adhesion	NA
ENSG00000258964	RP11-618G20.1	Chr14: 61,734,138-61,776,260 (+)	Unknown	Unknown	extracellular matrix organization	ECM-receptor interaction

## MATERIALS AND METHODS

### Data collection

The mRNA and lncRNA expression profile data of OvCa patients were obtained from the research of Du et al. [45] by repurposing the exon-array data on the Affymetrix Human 1.0 ST array from the Cancer Genome Atlas (TCGA) data portal (http://cancergenome.nih.gov/) [54]. Briefly, the probe sets of Human Exon 1.0 ST array were re-annotated to the human genome. Then those probes that uniquely mapped to lncRNA sequences were kept to represent lncRNAs. The expression levels of lncRNAs were obtained by background correction and quantile normalization [45]. The miRNA expression profile data of OvCa patients was downloaded from TCGA [54]. Finally, expression profiles of 18292 mRNA, 10207 lncRNA and 723 miRNA in 401 OvCa patients with stage information were included in our study.

Human miRNA and targets data were collected from TarBase (version 6.0) [55], miRTarBase (version 4.5) [56] and miRecords (version 4) [57], which provide high-quality experimentally validated miRNA-target interaction relationships manually curated from published experiments. By integrating the above three databases, a total of 37659 non-redundant miRNA-target interactions were used in our study. The experimentally validated miRNA-lncRNA interaction was downloaded from starBase v2.0 [58], including 10129 miRNA-lncRNA interactions.

Experimentally verified OvCa-related miRNAs, mRNAs and lncRNAs were obtained from HMDD [59], miR2Disease [60], miRCancer [61], NCG [62] and LncRNADisease [63] databases.

### Construction of lncRNA-associated ceRNA network

The lncRNA-associated ceRNA network was constructed based on “ceRNA hypothesis” as follows: First, expression correlation between mRNA and lncRNA was evaluated using Pearson correlation coefficient (PCC) from matched mRNA and lncRNA expression profiles data as follows:

$$\text{PCC}(\text{mRNA}\ln\text{cRNA})=\frac{1}{n-1}\sum_{i-1}^{n}\left(\frac{\text{Exp}(\text{mRNAi})-\bar{\text{Exp}(\text{mRNA})}}{\sigma (\text{mRNA})}•\frac{\text{Exp}(\ln\text{cRNAi})-\bar{\text{Exp}\left(\ln\text{cRNA}\right)}}{\sigma (\text{lncRNA})}\right)$$ (1)

Where n is the number of patients with OvCa; *Exp*(*mRNA,i*) (*Exp*(ln*cRNA,i*)) is the expression value of mRNA (lncRNA) in the OvCa patient i. $\bar{\text{Exp}(\text{mRNA})}(Exp(lncRNA,i))$ is the average expression level of mRNA (lncRNA), and σ(*mRNA*)(σ(ln*cRNA*)) denotes the standard deviation of expression level of mRNA (lncRNA). To reduce false positives, only top correlated mRNA-lncRNA pairs, whose correlation coefficient are higher than the threshold of the 99th percentile of the corresponding overall correlation distribution (Pearson correlation coefficient > 0.33) [11], were chosen for further analysis. Second, an lncRNA-mRNA pair in which both are positively correlated and interact with more than one same miRNA was considered as a candidate LMceCT. Third, the Pearson correlation coefficient for miRNA-mRNA and miRNA-lncRNA was computed using paired miRNA, mRNA and lncRNA expression profile data according to the above equation (1). If both mRNA and lncRNA in the same candidate LMceCT are co-expressed negatively with a certain common miRNA, this candidate LMceCT was identified as the functional LMceCT. Finally, all the functional LMceCTs were integrated to form a miRNA-mediated lncRNA-associated ceRNA network (LCeNET).

### Network analysis

The topological features of LCeNET, including degree, characteristic path length (CPL), betweenness centrality (BC), clustering coefficient (CC) and small world property (SWP), were analyzed. The degree of a node is the number of edges connecting to other nodes. The CPL of a network is the average shortest path length for all pairs of nodes. Lower CPL implies a more compact network form. The BC is an indicator of measuring the influence of a node exerting over the spread of information through the network. The high BC represents the key role of a node in communication and information diffusion [64]. The CC of a node measures the local cohesiveness, and the CC of network is the average of the CCs for all nodes in the network. The SWP can be calculated as follows [65]:

$$\sigma_{\text{SW}}=(\text{CC}/{\text{CC}}_{r})/(\text{CPL}/{\text{CPL}}_{r})$$ (2)

Where *CC*_r_ and *CPL*_r_ are respectively the CC and CPL of the corresponding random network. A network has ‘small word' property if the small-world index σ*_SW_*is larger than random network.

To determine the statistical significance of topological features, randomization test was performed by comparing real topological features with those of 1000 random network that preserve the same number of nodes and edges and keep the same degree of each node as in LCeNET. The empirical p-values of each measure were defined as the fraction of corresponding topological feature in 1000 random conditions which is greater than the value in the real condition. The comparison on attributes of network between LCeNET and random network was performed by using the R package “igraph”. The LCeNET was visualized using Cytoscape 3.2.0, and the functional modules were mined using MCODE algorithm which can effectively dig out densely connected regions of a molecular interaction network [21].

### Functional enrichment analysis

Functional enrichment analysis at the GO and KEGG levels was performed using DAVID Bioinformatics Resources (http://david.abcc.ncifcrf.gov/, version 6.7) [66]. The DAVID enrichment analysis was limited to KEGG pathways and GO- FAT biological process (BP) terms with the whole human genome as background. Functional categories with *p*-value of < 0.05 and an enrichment score of > 1.5 were considered statistically significant, and were visualized and clustered based on similar functions using the Enrichment Map plugin in Cytoscape 3.2.0 [67].

### Survival analysis

By fitting prognostic lncRNA biomarkers in a multivariate Cox regression analysis, a risk score model was constructed by considering the power of each of the prognostic lncRNA biomarkers as follows:

$$\text{Risk score}=\sum_{:}^{N}W_{i}\times {\text{Exp}}_{i}$$ (3)

Where *N* is the number of prognostic lncRNAs, *Exp_i_* is the expression level of prognostic lncRNA *i* and *W_i_* is the estimated regression coefficient of lncRNA *i* in the multivariate Cox regression analysis. The median value of risk score was chosen as the cutoff to classify patients with OvCa into high-risk group and low-risk group. Kaplan-Meier survival analyses were carried out to assess the difference in OS between high-risk group and low-risk group, and statistical signifi­cance was evaluated using the two-sided log-rank test using the R package “survival”. In addition, multivariate Cox regression analysis and data stratification analysis were performed to access whether the risk score model was independent of other clinical features. The time-dependent receiver operating characteristic (ROC) curve analysis was also performed to evaluate the sensitivity and specificity of risk score model for survival prediction using the R package “survivalROC”. Area under the curve (AUC) value was calculated from the ROC curve. All analyses were performed using R software and Bio-conductor.

## SUPPLEMENTARY MATERIAL FIGURES AND TABLES










