# Supplementary material for: Characterization of long non-coding RNA-associated ceRNA network to reveal potential prognostic lncRNA biomarkers in human ovarian cancer
Source: Oncotarget. 2016 Feb 3;7(11):12598–611. doi: 10.18632/oncotarget.7181 (PMC4914307; doi:10.18632/oncotarget.7181)
Supplement: Supplementary file 1 [file oncotarget-07-12598-s001.pdf]

Characterization of long non-coding RNA-associated ceRNA network to reveal potential prognostic lncRNA biomarkers in human ovarian cancer

Supplementary information

Supplementary file 1. List of 1270 miRNA-mediated lncRNA-mRNA ceRNA interactions in OvCa.
Supplementary file 2. List of enriched GO terms and KEGG pathways of mRNAs in **the LCeNET.**

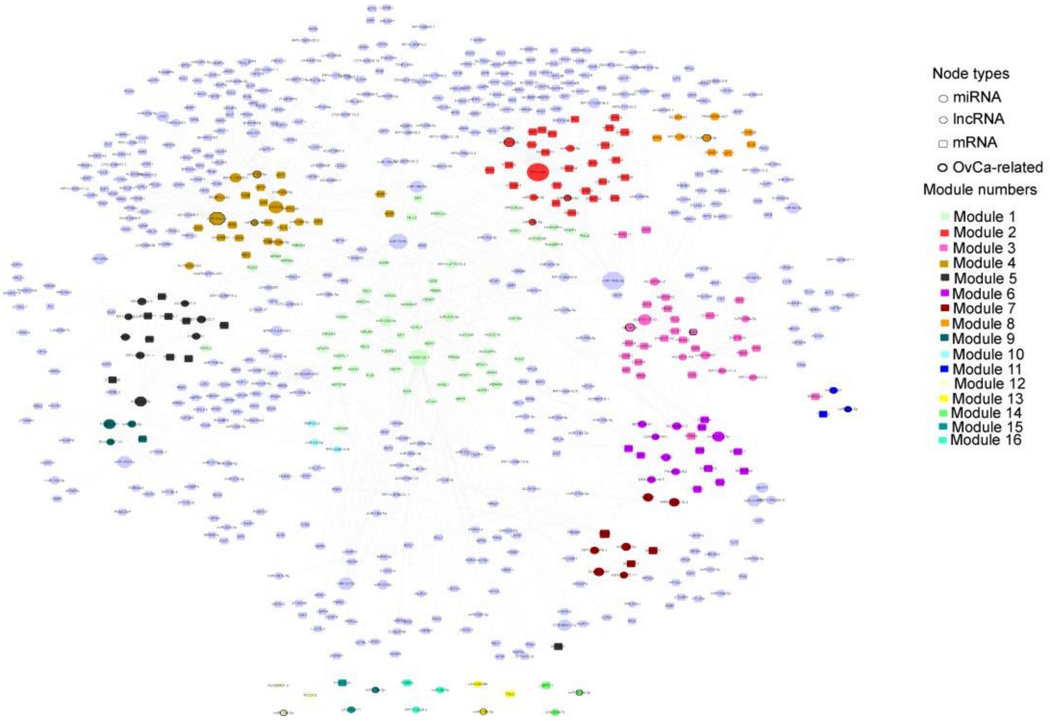

Supplementary file 3. Global view of the identified 16 modules in the **LCeNET.**
Supplementary file 4. List of miRNA-mediated lncRNA-mRNA ceRNA interactions in three stage-specific **LCeNETs.**

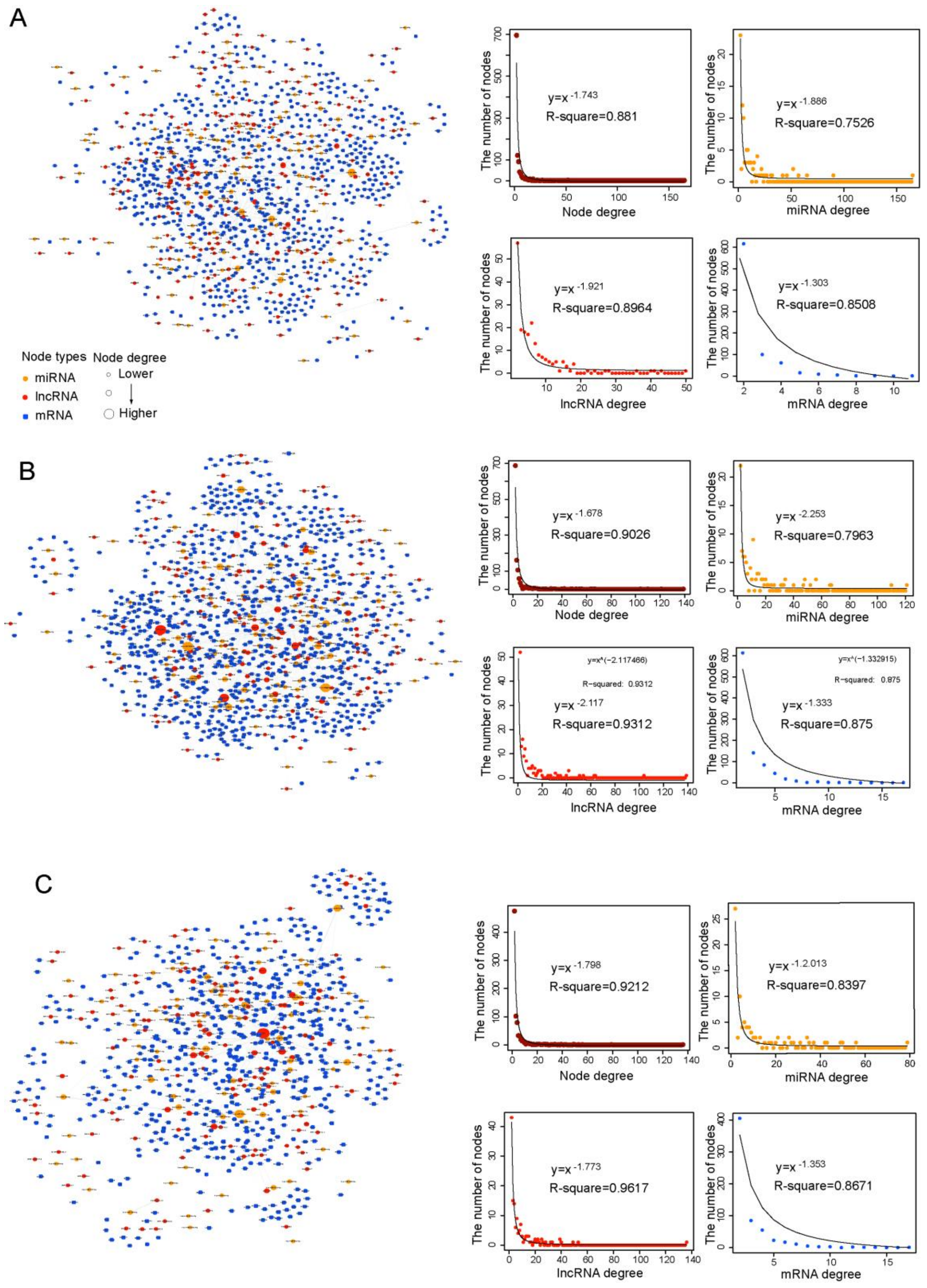

Supplementary file 5. Global view of **stage-specific LCeNETs and their topological characteristics.**

Supplementary file 6. List of hubs in three **stage-specific LCeNETs.**
